# Extracellular Vesicle-Derived circITGB1 Regulates Dendritic Cell Maturation and Cardiac Inflammation via miR-342-3p/NFAM1

**DOI:** 10.1155/2022/8392313

**Published:** 2022-05-16

**Authors:** Jianbing Zhu, Zhaoyang Chen, Xiaoping Peng, Zeqi Zheng, Aiping Le, Junjie Guo, Leilei Ma, Hongtao Shi, Kang Yao, Shuning Zhang, Junbo Ge, Zhenzhong Zheng, Qian Wang

**Affiliations:** ^1^Department of Cardiology, The First Affiliated Hospital of Nanchang University, Nanchang, Jiangxi Province 330006, China; ^2^Jiangxi Hypertension Research Institute, Nanchang, China; ^3^Heart Center of Fujian Province, Union Hospital, Fujian Medical University, 29 Xin-Quan Road, Fuzhou 350001, China; ^4^Department of Blood Transfusion, The First Affiliated Hospital of Nanchang University, Nanchang, Jiangxi Province 330006, China; ^5^Department of Cardiology, The Affiliated Hospital of Qingdao University, Qingdao, China; ^6^Department of Cardiology, Zhongshan Hospital, Fudan University, Shanghai, China

## Abstract

Acute myocardial infarction (AMI) is a complication of atherosclerosis-related cardiovascular illness that is caused by prolonged ischemia. Circular RNAs (circRNAs) are concentrated in extracellular vesicles (EVs) and have been linked to cardiovascular disease. However, additional research is needed into the expression and function of circRNAs in AMI. In this study, circITGB1 (has_circRNA_0018146), derived from exon 1 of the ITGB1 gene localized on chromosome 10, was shown to be considerably increased in plasma from patients with AMI compared to healthy controls, as demonstrated by the comparison of EV-circRNA expression patterns. Using a luciferase screening assay and a biotin-labeled circITGB1 probe to identify microRNA(s) complementary to circITGB1 sequences, we discovered that circITGB1 competitively binds to miR-342-3p and inhibits its expression, which in turn increase the expression of NFAT activating molecule 1 (NFAM1). Based on western blotting and immunological studies, circITGB1 controls dendritic cell maturation by targeting miR-342-3p and NFAM1. circITGB1 also exacerbated cardiac damage and regulated miR-342-3p and NFAM1 expression in a mouse AMI model. This implies that EV-circITGB1 is involved in dendritic cell maturation and cardiac damage via miR-342-3p/NFAM1, and that is linked to AMI-associated pathogenic processes.

## 1. Introduction

Despite significant advances in clinical care over the last two decades, cardiovascular diseases (CVDs) remain a leading cause of mortality in the developed world [1]. CVD risk appears to differ by area, with one study revealing that China had the highest CVD risk among the countries studied [2]. In China, the 10-year risk of fatal CVDs is over 10% in 28–33% of men and women compared to 5-10% of men and women in low-risk countries such as South Korea, Spain, and Denmark [2]. Although the reasons behind the disparities are unclear, research indicates that genetic background may be a factor because Chinese Han populations have genetic variants that are associated with susceptibility to CVDs [3].

Acute myocardial infarction (AMI) contributes to the detrimental effects of CVDs on the myocardium [4, 5]. Early restoration of blood flow to the ischemic myocardium is the most efficient treatment for AMI; nonetheless, myocardial reperfusion can trigger cardiomyocyte death and contribute to acute myocardial damage [6, 7]. A number of studies have demonstrated that certain factors can influence the detrimental effects of myocardial injury including oxidative stress caused by the generation of reactive oxygen species, increased intracellular and mitochondrial calcium levels, and inflammation [8–12]. The inflammatory response triggered by AMI has also been implicated in the pathogenesis of postinfarction remodeling and heart failure [13, 14]. Dendritic cells (DCs) are hypothesized to play a key role in the immunoprotective regulation of monocyte and macrophage homeostasis throughout the postinfarction healing process [14]. Moreover, mature activated DCs are infiltrated into the infarcted heart, and treatment of left ventricular remodeling following AMI may benefit from suppressing DC-mediated immune responses [15, 16]. It has been proposed that patients with overactive postinfarction inflammation might benefit from antichemokine therapies [17]. Therefore, a biomarker-based approach to identify these distinct pathophysiologic responses and to implement inflammation-modulating strategies has been suggested [12].

Circular RNAs (circRNAs) are a large class of noncoding RNAs, characterized by a closed loop structure, that are involved in the physiology and pathology of biological systems [18]. circRNAs are hypothesized to play a role in the development of cardiovascular disease and have been proposed as biomarkers for AMI [19–23]. Recent research discovered that the circRNA antisense to cerebellar degeneration-related protein 1 transcript (CDR1as or ciRS7) is upregulated in cardiomyocytes under hypoxia treatment and promotes AMI by interacting with miR-7a to prevent it from protecting cardiomyocytes [24]. Therefore, circRNAs are thought to compete for endogenous RNA to reduce the capacity of miRNAs and deregulate the expression of miRNA target genes [25–27].

Extracellular vesicles (EVs) are complex membranous structures composed of a lipid bilayer and include exosomes, microvesicles, and apoptotic bodies. Exosomes are nanometer-sized vesicles released from cells into the blood, which are enriched in circRNA [28]. In recent years, exosomes have been regarded as therapeutic delivery agents because they are tolerated by the immune system and are natural nanocarriers derived from endogenous cells [29]. In the present study, we compared the plasma EV-derived-circRNA expression profiles of patients with AMI to controls. We discovered that circITGB1 (hsa_circRNA_0018146) was significantly upregulated in plasma EVs from patients with AMI. We used luciferase screening assay and a biotin-labeled circITGB1 probe to pull down microRNAs complementary to the circITGB1 sequence and further analyzed the influence of these interacting sequences on target miRNA. This allowed us to identify that circITGB1 interacts with miR-342-3p, which is associated with NFAT activating molecule 1 (NFAM1), a protein involved in the innate immune response and DC activation in healthy participants who received the yellow fever-17D vaccination [30]. When DCs are activated upon stimulation, they undergo maturation and acquire an enhanced capacity to form and accumulate peptides, major histocompatibility complex class II molecules, costimulatory molecules (such as CD80 and CD86), and antigens (such as CD83) [31]. In this study, we used the DC maturation markers CD80, CD86, and CD83 to investigate whether circITGB1 may be involved in DC maturation. Finally, we analyzed the influence of overexpressing circITGB1 in a mouse model of AMI.

## 2. Methods

### 2.1. Patients

To screen candidate circRNAs, 20 consecutive AMI patients and 20 healthy volunteers were recruited in Shanghai Zhongshan Hospital between September 2016 and May 2017. Plasma samples from 20 AMI patients and 20 healthy volunteers were collected and examined to validate the candidate circRNAs. Subjects were diagnosed with AMI using the following criteria: (1) coronary angiography indicated at least one major epicardial coronary artery with a 50% stenosis, (2) chest pain lasting >20 mins, (3) pathological Q waves or ST-segment elevation/depression on the electrocardiogram (ECG), and (4) elevation of traditional myocardial markers. Patients were excluded if they had received intravenous thrombolytic or anticoagulant therapy before the initial blood samples were obtained, as well as patients with a confirmed diagnosis of impaired ejection fraction or heart failure. 20 adult healthy volunteers (normal ECG finding and no history of CVDs, 10 men, 10 women; 60.7 ± 7.3 years) selected as controls were matched by age, sex, and area of residence with the patients. About 5 mL of venous blood samples were collected from each participant in EDTA-anticoagulant tubes. The blood samples of patients with AMI were collected within 24 hours of the chest pain, and the average period of time from symptom onset to blood sampling was 6.9 ± 2.2 hours. Plasma was isolated from the whole blood samples by centrifugation (×g) for 15 mins and was stored at -80°C.

The protocol of this study was carried out according to the principles of the Declaration of Helsinki and approved by the Medical Ethics Committee in Shanghai Zhongshan Hospital. Written informed consent was obtained from all the participants before enrolment. The baseline clinical characteristics of patients and controls are presented in Table 1.

### 2.2. Extraction of EVs from Plasma

EVs were purified from plasma samples using ExoQuick® precipitation solution (System Biosciences, Palo Alto, CA, USA) according to the manufacturer's instructions. Briefly, plasma samples were first filtered through 0.22 *μ*m pore filters, then mixed with an ExoQuick® reagent and incubated at 4°C for 30 mins to precipitate the EVs. EVs were then pelleted by centrifugation at 1500 × *g*, 4°C, for 30 mins, then resuspended in nuclease-free water. Transmission electron microscopy (TEM) and fluorescence-activated cell sorting (FACS) analysis of the surface proteins CD63, CD81, and Hsp70 were used to identify and verify EVs.

### 2.3. Total RNA Preparation

Total RNA was isolated from plasma EVs using a TRIzol kit (Invitrogen, Carlsbad, CA, USA) and miRNeasy Micro kit (QIAGEN, Valencia, CA, USA) in accordance with the manufacturer's instructions. The purity and concentration of the total RNA in each sample were measured using a BioMate 3S spectrophotometer (Thermo Scientific, Wilmington, DE, USA).

### 2.4. Microarray Processing

Global profiling of human circRNAs was performed using an Arraystar Human circRNA Microarray, version 2.0 (Arraystar Inc., Rockville, MD, USA). The samples were prepared and hybridized to the array in accordance with the manufacturer's instructions. Briefly, total RNA was digested with RNase R (Epicentre, Inc., Madison, WI, USA) to remove linear RNAs. The enriched circRNAs were then amplified and hybridized. circRNAs which were differentially expressed between the two samples were identified by fold-change filtering. Hierarchical clustering was used to reveal distinguishable circRNA expression pattern in samples.

### 2.5. Quantitative RT-PCR

Quantitative RT-PCR was used to analyze circRNAs and miR-342-3p. Reactions were performed on a CFX96 Detection System (Bio-Rad, Hercules, CA, USA) using an RT Kit (Takara, Dalian, China) and PCR Master Mix (Takara, Dalian, China) in accordance with the manufacturer's instructions. Briefly, random primers were used to reverse-transcribe 500 ng of total RNA into cDNA, and 10 ng of cDNA was used for real-time PCR. The reactions were performed in a 96-well optical plate, and the conditions were as follows: predenaturation at 95°C for 30 s, followed by 40 cycles of PCR (denaturation at 95°C for 5 s, annealing at 60°C for 20 s). Primer sequences used in qPCR are as follows: circITGB1, 5′-TCGGGACAAATTACCCCAGC-3′ (forward) and 5′-GTTGCACTCACACACACGAC-3′ (reverse); miR-342-3p, 5′-GGGTCTCACACAGAAATCGC-3′ (forward) and 5′-CAGTGCGTGTCGTGGAGT-3′ (reverse); GAPDH, 5′-GGAGCGAGATCCCTCCAAAAT-3′ (forward) and 5′-GGCTGTTGTCATACTTCTCATG-3′ (reverse); and U6, 5′-GATTATCGGGACCATTCCACTG-3′ (forward) and 5′-GATCTGGTTCCCAATGACTGTG-3′ (reverse). GAPDH was used as the endogenous control for circRNA qPCR and U6 for miRNA. The primers used for the qPCR assay for other genes are listed in Table S1.

### 2.6. EV Characterization

Pellets from plasma containing EVs were resuspended in 0.2% paraformaldehyde and placed on formvar-carbon-coated nickel electron microscopy grids (Electron Microscopy Sciences, Hatfield, PA, USA). The samples were stained with 1.75% uranyl acetate and then observed under a transmission electron microscope (Hitachi Ltd., Tokyo, Japan). The sizes of EVs were determined using a NanoSight LM10 device (NanoSight, Amesbury, UK).

### 2.7. Nanoparticle Tracking Analysis (NTA)

NTA was used to determine the EVs' concentration and size distribution. Briefly, the EV samples were diluted in phosphate-buffered saline (PBS, 200x and 1000x) and examined with the NanoSight LM10/LM14 apparatus (NanoSight Ltd., Malvern, UK) (*n* = 6). A syringe pump was used to inject the samples automatically for each capture, three 60-second movies were made for each dilution, and the analysis was done with NanoSight NTA 3.1 software.

### 2.8. Monocyte-Derived Dendritic Cells

Ficoll density centrifugation was used to isolate peripheral blood mononuclear cells (PBMCs) from the whole blood [32]. Briefly, a positive selection kit (Miltenyi Biotec, Auburn, CA, USA) was used to isolate and purify the CD14+ monocytes from the PBMCs. The purified monocytes were cultured at a density of 1 × 10^6^/mL for 6 days, in RPMI 1640 medium supplemented with 10% FCS, 2 mM l-glutamine, 100 units/mL penicillin, 100 g/mL streptomycin, granulocyte-macrophage colony-stimulating factor (R&D Systems, Minneapolis, MN, USA; 30 ng/mL), and IL-4 (R&D Systems, Minnesota, MN, USA; 20 ng/mL). Maturation was induced in immature monocyte-derived DCs (mdDCs) with lipopolysaccharide (LPS, Sigma-Aldrich, St Louis, MO, USA; 100 ng/mL). Changes in costimulatory and maturation markers were analyzed by flow cytometry.

### 2.9. Adenoviral Constructions and Infection

The circRNA ITGB1 vector was synthesized as described previously [26, 33]. The circITGB1 exon along with the endogenous flanking sequence (1 kb upstream) was inserted into pcDNA3.1, and then, part of the upstream flanking sequence was copied and inserted in an inverted orientation downstream. circITGB1-ir without the downstream reverse sequence was used as a negative control. All vectors were finally cloned into the Adeno-X Expression System (Clontech, Otsu, Japan) according to the manufacturer's instructions.

Small interfering RNAs (siRNAs) targeting human circITGB1 (circITGB1 siRNA) and control siRNA were obtained from GenePharma Co. Ltd. (Shanghai, China). NFAM1 siRNA and a scramble siRNA (NFAM1-sc) were purchased from Santa Cruz Biotechnology (Dallas, TX, USA). The chemically modified antagomir complementary to miR-342-3p designed to inhibit endogenous miR-342-3p expression and the antagomir negative control (anti-miR-control) were obtained from GenePharma Co. Ltd. (Shanghai, China). Transfection was performed using Lipofectamine 2000 following the manufacturer's instructions.

### 2.10. Multiplex Assay Kits

Human TNF-*α*, IL-12p70, IL-1*β*, and IL-6 were measured in culture supernatant using the V-Plex MSD electrochemiluminescence multispot assay platform (Meso Scale Diagnostics, Rockville, MD, USA). Each plate was imaged on the Meso Quickplex SQ 120 (Meso Scale Discovery, K15067L-2), following the manufacturer's instructions. A SECTOR® Imager 6000 device was used to read the plates. The Discovery Workbench 3.0 software was used to examine the data. All samples were assayed in duplicate.

### 2.11. Bead-Based Flow Cytometric Analysis

Briefly, EV pools were identified by bead-based flow cytometry assessing the presence of CD63, CD81, and HSP70. For each sample, 10 *μ*g of EVs was incubated with 100,000 beads (3.5 × 10^8^/mL; 4 *μ*m aldehyde/sulphate latex beads; Thermo Fisher) overnight with gentle agitation. The bead-EVs were washed with BCB buffer and resuspended in PBS after a second centrifugation at 2000 × *g* for 10 mins. EV-coated beads were then labelled at 4°C with CD63-Alexa Fluor 488 (MEM-259, Thermo Fisher), anti-CD81-PE-conjugated (A15781, Thermo Fisher), anti-HSP70-PE (ab208878, Abcam, UK), and polyclonal IgG isotype (IgG1-PE MA1-10415, IgG1-FITC MA1-10413, Thermo Fisher) antibodies for 30 mins at room temperature. The bead-EVs were washed three times and acquired on a FACSVerse (BD Bioscience, San Jose, CA, USA). The data were analyzed using FlowJo™ 10 (FlowJo, LLC, Ashland, OR, USA). Each measurement was performed in technical triplicates. In total, 10,000 events/sample were analyzed.

### 2.12. Treatment with 5-(N,N-Dimethyl)-amiloride Hydrochloride (DMA)

DMA was purchased from Sigma-Aldrich (St. Louis, MO); human mdDCs were pretreated with DMA (21.3 mM) for 1 h prior to overnight stimulation with LPS (100 ng/mL). Cells were collected for follow-up experiments.

### 2.13. Western Blotting Analysis

Samples were first applied to a 12% Tris-HCl gel and then transferred to nitrocellulose membranes (Millipore, Billerica, MA, USA). Membranes were then blocked with 5% skim milk or 5% BSA in 0.05% Tween 20-TBS (Tris-buffered saline) (USB Corporation, Fremont, CA, USA) for 1 h and incubated with a primary antibody anti-CD63 (ab68418, Abcam, 1 : 1000), anti-CD81 (ab155760, Abcam, 1 : 1000), anti-Hsp70 (ab5439, Abcam, 1 : 1000), and anti-NFAM1 (bs-2680R, Bioss, 1 : 500) overnight at 4°C and then detected with a secondary antibody (7074S, Cell Signaling) for 1 h at room temperature. The protein bands were visualized with enhanced chemiluminescence and normalized to *β*-actin. Images were processed using ImageJ version 1.41 (National Institutes of Health, Bethesda, MD, USA).

### 2.14. Luciferase Reporter Assay

The complementary sequences between circITGB1 and miRNA (miR-342-3p, miR-329-3p, miR-377-3p, miR-149-5p, and miR-362-3p) were displayed by starBase (http://starbase.sysu.edu.cn/). The binding regions of miR-342-3p within NFAM1 were predicted by TargetScan (https://www.targetscan.org/vert_80/). The HEK293T (293T) cell line was purchased from the American Type Culture Collection (ATCC) and cultured in Dulbecco modified Eagle medium (DMEM) supplemented with 10% fetal calf serum (FBS). HEK293A (239A) (Thermo Fisher Scientific, San Jose, CA, USA, R70507) cell lines were cultured in DMEM supplemented with 10% FBS.

The wild-type (wt) and mutant-type (mut) fragments of circITGB1 and wt and mut type 3′-UTR of NFAM1 were synthesized and constructed into the pGL3 reporter vector (Promega Madison, WI, USA). After reaching 70-80% confluence, 293T cells were cotransfected with 50 nmol/L miR-342-3p mimic or control (Gene pharma; Shanghai), circITGB1-wt (100 ng/well), or circITGB1-mut (100 ng/well) luciferase reporter plasmid and 10 ng pRL-TK (expressing Renilla luciferase as the internal control) using the Lipofectamine® 2000 Transfection reagent (Life Technologies, Carlsbad, CA, USA). The 293A cells were cotransfected with 100 ng NFAM1-wt or mut luciferase reporter constructs, 10 ng pRL-TK (expressing Renilla luciferase as the internal control) (Promega Corporation), and 20 nM miR-342-3p mimic or control by using the Lipofectamine® 2000 Transfection reagent. After 48 h of transfection, the luciferase activities were determined with a Dual Luciferase Reporter System (Promega Corporation) and normalized to Renilla luciferase activity. All constructs were evaluated in a minimum of three separate experiments.

### 2.15. Biotin Pull-Down of RNA

To detect circITGB1 and miR-342-3p interactions, a biotin pull-down assay was carried out. qPCR was used to detect miR-342-3p- or circITGB1-enriched human mdDC lysates using a biotinylated-oligonucleotide probe for both circITGB1 and miR-342-3p. The mdDCs were cross-linked with 1% formaldehyde in PBS for 10 mins at room temperature, then quenched with 0.125 M glycine for 5 mins. The cells were resuspended in a lysis buffer on ice for 10 mins and sonicated. The cell lysate was then diluted in two volumes of hybridization buffer. Next, 100 pmol biotin probes were added. Streptavidin Dynabeads (Life Technologies) were blocked for 2 h at 4°C in lysis buffer containing 1 mg/mL yeast tRNA and 1 mg/mL BSA and washed twice with 1 mL lysis buffer. Then, 100 *μ*L washed/blocked Dynabeads were added per 100 pmol of biotin probes and rotated for 30 mins at 37°C. Beads were captured using magnets (Life Technologies) and washed five times. RNA was then eluted from the beads with a buffer (Tris 7.0, 1% SDS).

### 2.16. Fluorescence In Situ Hybridization

Cultured human mdDCs were fixed in 4% paraformaldehyde for 5 mins at room temperature and then washed with PBS. *In situ* hybridization was performed on samples with specific probes for circITGB1 and miR-342-3p using a miRCURY LNA microRNA ISH Optimization Kit (QIAGEN, Hilden, Germany) following the manufacturer's instructions. Fluorescence-labeled probes in a hybridization buffer were incubated with samples at 55°C for 1 h. DAPI (157574, MB Biomedicals) was used for nuclear counterstaining. Images were captured using confocal microscopy (DM6000 CFS, Leica, Switzerland) and processed with LAS AF software.

### 2.17. Animal Experiments

All animal experiments were authorized by the Institutional Animal Care and Use Committee of Zhongshan Hospital at Fudan University on July 19, 2016. Adult male C57BL/6 mice (purchased from the Shanghai Laboratory Animal Center) were utilized in this study and kept in pathogen-free conditions. Twelve-week-old male C57BL/6 mice were used as donors for the culture of bone marrow-derived dendritic cells (BMDCs), while eight-week-old male C57BL/6 mice were used to generate a model of AMI and then injected with EVs. BMDCs were isolated from the C57BL/6 mice according to a previously reported method [31]. Cultured BMDCs were pretreated with circITGB1-ir or circTGB1 for 1 h prior to overnight stimulation without (control) or with LPS. The BMDC supernatants were collected for EV isolation using the ExoQuick-TC™ Exosome Precipitation Solution according to the manufacturer's protocol (System Biosciences).

### 2.18. AMI Model and Injection of EVs

The induction of the AMI was performed as described previously [34]. Briefly, following anesthetization with 2% isoflurane, the heart was exposed through a small chest incision, and the left main descending coronary artery (LCA) was located and permanently ligated with a 7-0 silk suture 2-3 mm from the origin of the left atrium. Echocardiography was performed before and 4 weeks after the procedure to validate the efficacy of the ligations. To analyze the effect of EVs on myocardial tissue, we suspended EVs (400 *μ*g of protein) from different groups in 200 *μ*L of PBS and administered them via the tail vein immediately after the ligation operation. Animals were then followed for 4 weeks and then euthanized. Cardiac muscle tissues were collected for analyses.

### 2.19. Echocardiography

At 4 weeks after AMI, *in vivo* cardiac function was determined by echocardiography (Vevo 2100, VisualSonics, Toronto, Canada). Mice were first anesthetized with 2% isoflurane and oxygen. Two-dimensional echocardiographic views were obtained of the left ventricular (LV) long axis through the anterior and posterior LV walls below the mitral valve and level with the papillary muscle tips. Echocardiography was evaluated by calculating the LV ejection fraction (LVEF) and fractional shortening (FS) and measuring the left ventricular end-diastolic diameter (LVDd) and end-systolic diameter (LVDs) in a blinded manner.

### 2.20. Quantification of Myocardial Infarct Size

After 4 weeks, mice were euthanized with an injection of KCl and hearts were collected for infarct size (IS) analyses using 2,3,5-triphenyltetrazolium (TTC). In brief, LVs were isolated and cut into 4 mm slices perpendicular to the axis of the LCA and placed on slides. To distinguish infarct tissue from the healthy myocardium, the sections were immediately stained with 1% TTC in phosphate buffer (pH 7.4) at 37°C for 15 min [35]. The IS was calculated as a percentage of the LV with the use of digital planimetry software (Image-Pro Plus 6.0).

### 2.21. Hematoxylin-Eosin (H&E) Staining

The structure of mouse cardiac tissue was examined using H&E staining. In order to preserve the hearts of mice, we used 4% paraformaldehyde to fix them. It was then converted into paraffin blocks. A microtome was used to cut paraffin blocks into 5 *μ*m thick slices. We utilized xylene and gradient alcohol for dewaxing and hydration prior to the H&E staining. Hematoxylin (Beyotime, Shanghai, China) was employed to stain the cell nucleus, and hydrochloric acid alcohol was utilized to differentiate the cells. Our next step was to stain the cells' cytoplasm with eosin (Beyotime, Shanghai, China). Myocardial tissue was then dried with alcohol and mounted using a neutral mounting medium.

### 2.22. Statistical Analysis

All of the data are presented as the means ± SD. Differences between two groups were assessed with Student's *t*-test. Statistical analysis among more than two groups was conducted with one-way analysis of variance (ANOVA) followed by the Tukey post hoc test. A value of *P* < 0.05 was considered statistically significant. Statistical analysis was performed using GraphPad Prism 8 software (GraphPad Software, San Diego, CA, USA).

## 3. Results

### 3.1. Plasma EV-circRNA Profiles in Patients with AMI versus Controls

**Figure 1:**
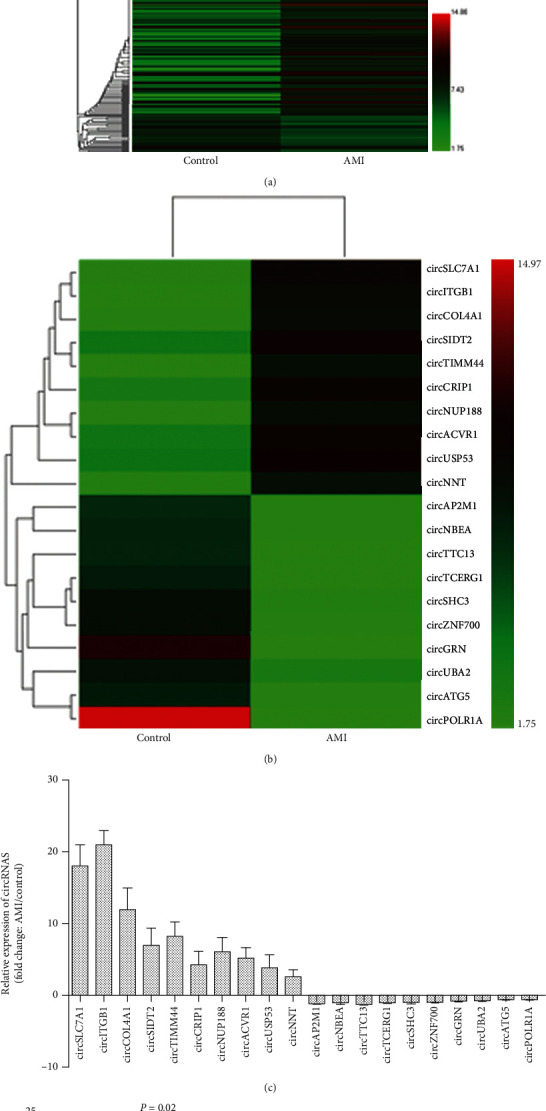
Expression profiles of EVs-circRNAs in patients with AMI and controls. (a) circRNA microarray identified dysregulated circRNAs between AMI patients and healthy controls. Hierarchical clustering of EV-circRNA expression profiling among samples. RNA was isolated from the pooled plasma of controls (*n* = 20) and patients with AMI (*n* = 20). Each row represents a circRNA. (b) The heat map illustrates the top 10 most upregulated and downregulated circRNAs in patients with AMI as compared to healthy controls analyzed by circRNA Arraystar Chip. “Red” indicates increased expression, and “green” indicates decreased expression. (c) Relative expression of 20 circRNAs measured by qPCR is shown with fold changes in two groups (*n* = 20/group) (increased and decreased). (d) Relative expression of the four indicated circRNAs (circSLC7A1, circITGB1, circATG5, and circPOLR1A) from 20 patients with AMI and 20 healthy controls listed in (c) measured by qPCR; GAPDH was measured as a reference gene. The data are presented as the means ± SD.

We compared the EV-circRNA profiles of patients with AMI and healthy controls using a circRNA microarray approach. Hierarchical clustering results demonstrate diverse circRNA expression profiles among the samples (Figure 1(a)).  Figure 1(b) depicts the circRNAs with the largest increases and decreases in expression between the AMI and healthy control groups. Thus, we established the first EV-circRNA expression profile of patients with AMI in our investigation. Following that, we confirmed the expression of the twenty differentially expressed circRNAs in EVs from patients with AMI and control samples from 20 patients by using qPCR (Figure 1(c)). circSLC7A1, circITGB1, circATG5, and circPOLR1A were the four most significantly changed circRNAs in Figure 1(d). In patients with AMI, circITGB1 expression was shown to be consistently and significantly elevated compared to matched healthy controls (Figure 1(d)). Thus, we focused on the expression and role of circITGB1 in the progression of AMI in this study.

### 3.2. circITGB1 Is Highly Enriched in EVs from the Plasma of AMI Patients

**Figure 2:**
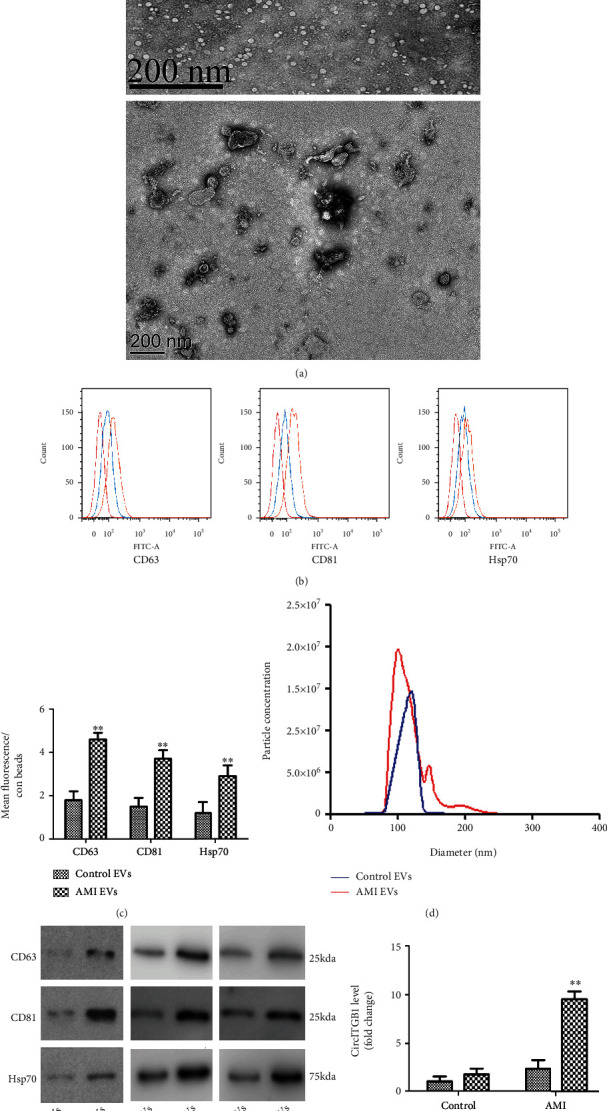
circITGB1 is highly enriched in EVs from the plasma of AMI patients. (a) Representative micrograph of EVs isolated from plasma of healthy control and AMI patients, as detected by TEM (*n* = 3). (b, c) Flow cytometry analysis of microspheres coated with EV-rich fractions isolated from plasma of healthy controls identified the presence of exosomal markers CD63, CD81, and HSP70, both at baseline (control, blue) and AMI (pink). Yellow curve indicates EV-coated microspheres stained with secondary antibodies (control). Collective analyses of FACS results in the two groups, *n* = 3. (d) NTA analysis of EVs derived from control and AMI plasma. (e) Western blots confirmed the presence of exosomal marker proteins CD63, CD81, and HSP70 in healthy control and AMI samples, *n* = 3. Levels of all proteins were increased in AMI. (f) Levels of circITGB1 in the EVs and supernatant fractions (Sup) of the plasma from patients with AMI and healthy controls, as detected by qPCR (*n* = 10 for each group); GAPDH was measured as a reference gene. Data are expressed as means ± SD. Statistical significance was calculated using Student's *t*-test or one-way ANOVA. ^∗∗^*P* < 0.01 compared with the control group.

The secreted EVs isolated from the plasma of five AMI patients at 24 h after chest pain onset were identified using TEM. Rounded structures with heterogeneous diameters were observed, most of which were between 40 and 100 nm, indicating that the isolated vesicles comprised many EVs (Figure 2(a)). The characteristics of the EVs were further confirmed with flow cytometry and western blotting. FACS analysis of the surface proteins CD63, CD81, and Hsp70, three commonly used markers of EVs, were detected in both control and AMI plasma-derived samples (Figure 2(b)). After normalization to control beads, the fluorescence expression of CD63, CD81, and Hsp70 in EVs isolated from AMI patients was higher compared with that from the control group (Figure 2(c)). Next, using nanoparticle tracking analysis (NTA), we were able to determine the size distribution of the separated particles (Figure 2(d)). Western blotting confirmed that CD63, CD81, and Hsp70 expression was increased in AMI patients compared to the control (Figure 2(e)), which suggests an elevated level of EVs in the plasma of AMI patients than healthy controls.

To investigate whether circITGB1 is released directly into the plasma or is protected within EVs, we separated plasma collected from AMI patients into EVs and supernatant fractions. The levels of circITGB1 were quantified in each fraction. The level of circITGB1 in the EVs was substantially higher than that in the supernatant fractions, which demonstrated that it was highly enriched in EVs (Figure 2(f)).

### 3.3. EV-circITGB1 Regulates Human mdDC Maturation

**Figure 3:**
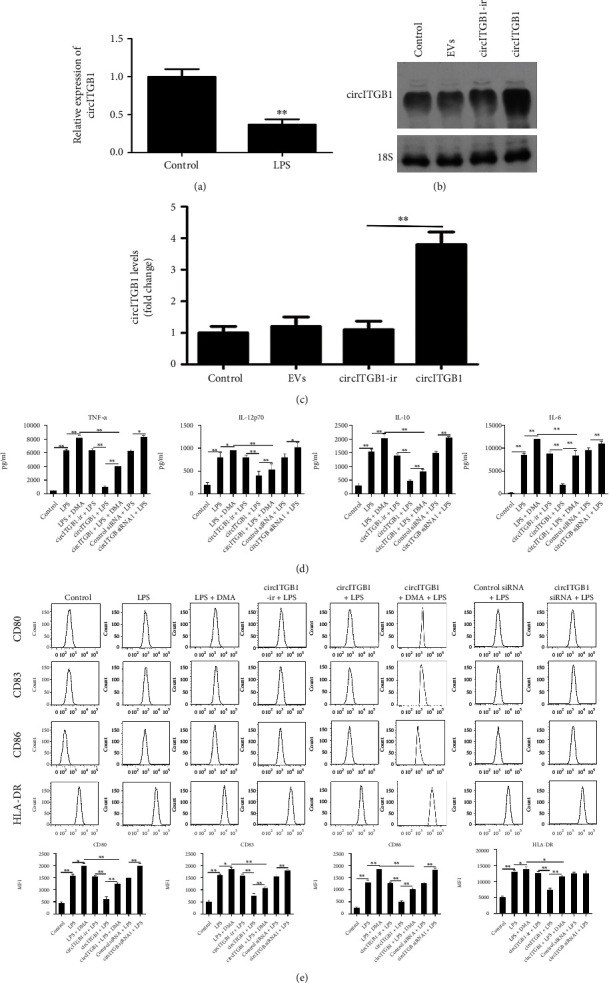
EV-circITGB1 regulates DC maturation. (a) circITGB1 expression in mdDC-EVs and human mdDCs was treated with 100 ng/mL LPS. Untreated mdDCs were used as control. RNA was extracted and circITGB1 expression was detected by qPCR. (b) Northern blot with 10 *μ*g of RNA from human mdDCs infected with adenovirus harboring empty vector, circITGB1-ir, or circITGB1. The blot is probed against circITGB1 and 18S ribosomal RNA (loading control), *n* = 3. (c) Human mdDCs were infected with adenovirus harboring empty vector, circITGB1-ir, or circITGB1; circITGB1 levels were analyzed by qPCR; and GAPDH was measured as a reference gene. (d) Cultured human mdDCs were pretreated with circITGB1-ir, circITGB1, control siRNA, circITGB1 siRNA, or the exosomal pathway inhibitor DMA (21.3 mM) for 1 h followed by overnight stimulation with LPS (100 ng/mL). Culture supernatants were assayed for TNF-*α*, IL-12p70, IL-1*β*, and IL-6. Values represent mean cytokine content in pg/mL ± SD from four independent mdDC cultures derived from four healthy donors. (e) Human mdDCs were pretreated with circITGB1-ir, circITGB1, control siRNA, circITGB1 siRNA, or DMA (21.3 mM) for 1 h prior to overnight stimulation with LPS (100 ng/mL). Cells were collected, stained, and analyzed for surface marker expression using flow cytometry. Histograms show expression of CD80, CD83, CD86, and HLA-DR on viable CD11c+ cells and is representative of *n* = 4 donors. Median fluorescence intensity of gated cells for corresponding surface markers ± SD, from 4 independent cultures. Significance was analyzed using Student's *t*-test or one-way ANOVA. ^∗^*P* < 0.05, ^∗∗^*P* < 0.01.

Our previous study indicated that exosomes released by DCs improve heart function following a AMI [36], and a more recent study has uncovered the role of circRNAs during DC formation and maturation [37]. We examined the necessity of EV-circRNA in regulating the functions of mdDCs in light of the increased circITGB1 expression found in 20 plasma EVs from AMI patients. As shown in Figure 3(a), EVs from mdDCs expressed circITGB1 and LPS, which is a stimulator for mdDC maturation, significantly reducing circITGB1 expression in DCs-EVs. Then, we stimulated immature mdDCs with LPS in the presence or absence of EV-circRNA. We used a vector-based system to overexpress circITGB1; circITGB1-ir was used as a negative control. These vectors were developed in an adenoviral system and used for further experiments. The overexpression of circITGB1 in human mdDCs was compared to control mdDCs and those infected with an adenovirus-harboring empty vector; circITGB1-ir was confirmed by northern blotting (Figure 3(b)) and qPCR (Figure 3(c)).

The cytokines TNF-*α*, IL-1*β*, IL-12p70, and IL-6 were measured in mdDCs with and without LPS stimulation in connection to circITGB1. LPS-stimulated human mdDCs were suppressed by the overexpression of circITGB1 while treatment with exosome release inhibitor DMA reversed this effect. The inhibition of circITGB1 or EV release increased the secretion of inflammatory mediators, including TNF-*α*, IL-1*β*, and IL-6, in the LPS-stimulated human mdDCs (Figure 3(d)).

Next, we analyzed the ability of mdDCs to mature upon LPS stimulation. Overexpression of circITGB1 during human mdDC maturation resulted in a considerably decreased expression of maturation markers CD80, CD83, CD86, and HLR-DR, and DMA treatment reversed the effects of circITGB1 overexpression. The inhibition of circITGB1 or EV release during the maturation of human mdDCs resulted in a significantly higher expression of the maturation markers CD80, CD83 and CD86, whereas HLR-DR was not significantly affected. These results indicate that EV-circITGB1 was involved in the regulation of the maturation of human mdDCs (Figure 3(e)).

### 3.4. circITGB1 Acts as a miR-342-3p Sponge to Regulate NFAM1 Expression in mdDCs

**Figure 4:**
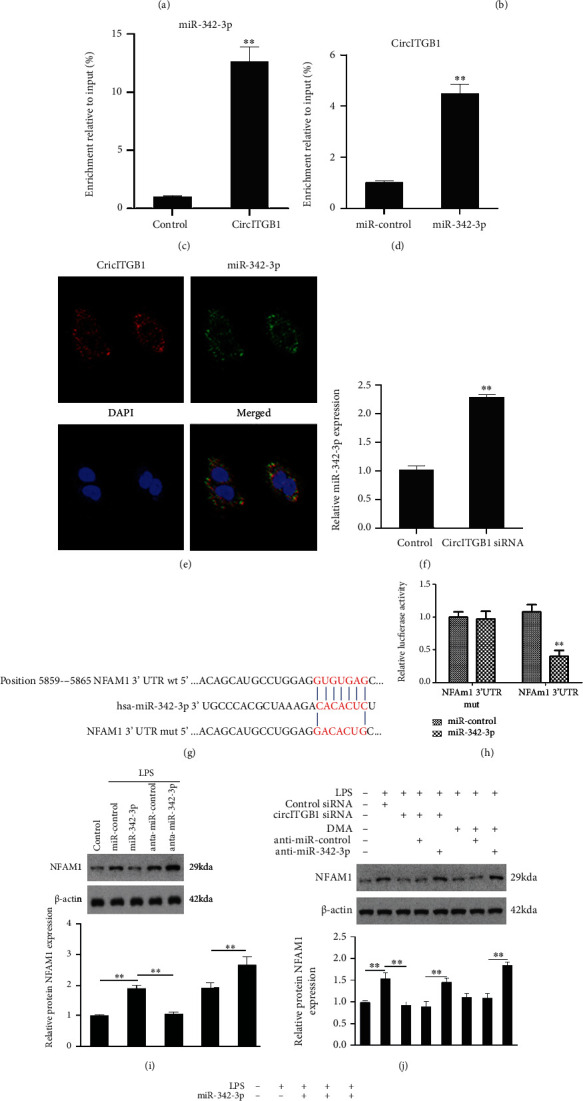
circITGB1 acts as a miR-342-3p sponge to regulate NFAM1 expression in mdDCs. (a) Prediction of miRNA binding sites in the circITGB1 sequence. (b) Luciferase reporter assay for the luciferase activity of circITGB1 or circITGB1-mut 293T cells cotransfected with five miRNA mimics or miR-control. Data are the means ± SD of three independent experiments. ^∗∗^*P* < 0.01 vs. miR-control+circITGB1 mut. (c, d) qPCR detected miR-342-3p (c) or circITGB1 (d) enriched from human mdDC lysates by biotinylated-oligonucleotide probe for circITGB1 or miR-342-3p. (e) Colocalization between miR-342-3p and circITGB1 was detected by RNA in situ hybridization. (f) The effect of circITGB1 knockdown on miR-342-3p expression was investigated by qPCR. (g) Predicted NFAM1 target region and miR-342-3p through TargetScan website. (h) Luciferase assay in 293A cells cotransfected with the indicated RNA or luciferase reporters. ^∗∗^*P* < 0.01 vs. miR-control+NFAM1 3′UTR mut. (i) Western blotting analyzed NFAM1 expression; *β*-actin was used as the loading control. (j) Western blotting analyzed NFAM1 expression in mdDCs transfected with the indicated RNA; *β*-actin was used as the loading control. (k) Western blotting analyzed NFAM1 expression; *β*-actin was used as the loading control. Significance was analyzed using Student's *t*-test or one-way ANOVA. ^∗^*P* < 0.05, ^∗∗^*P* < 0.01.

Because circRNAs are expected to work predominantly competitively, bind functional miRNAs, and then affect gene expression, we next examined the possibility that miRNAs associated with circITGB1 could regulate gene expression. The public database starBase, version 2.0 (http://starbase.sysu.edu.cn/) was used to screen for miRNAs that may be targeted by circITGB1. The search indicated that circITGB1 has sites where miR-342-3p, miR-329-3p, miR-377-3p, miR-149-5p, and miR-362-3p could be bound (Figure 4(a)). To identify the miRNAs that bind to circITGB1, we performed a luciferase screening assay. Each miRNA mimic was cotransfected with the luciferase reporters into 293T cells. Compared to the control RNA, only miR-342-3p had the capacity to reduce luciferase reporter activity by at least 50% (Figure 4(b)).

We conducted RNA pull-down assays with biotin-labeled circITGB1 probe or biotin-labeled miR-342-3p to further confirm the direct interaction between circITGB1 and miR-342-3p. The results from the qPCR analysis revealed that there was a 10-fold enrichment of miR-342-3p in the circITGB1-pulled down sediments (Figure 4(c)). Consistently, circITGB1 was also enriched in miR-342-3p-pulled down sediments (Figure 4(d)). The results of RNA in situ hybridization were consistent with interactions between circITGB1 and miR-342-3p in human mdDCs (Figure 4(e)). Furthermore, qPCR results revealed that circITGB1 knockdown resulted in a significant increase in miR-342-3p expression (Figure 4(f)). These results also suggest that circITGB1 may function as a sponge for miR-342-3p.

In further investigations, we discovered that the 3′-UTR of human NFAM1 contains a miR-342-3p-binding site at nucleotides 5859-5865 (Figure 4(g)). In a luciferase assay, the miR-342-3p mimic decreased NFAM1-3′-UTR-directed luciferase activity by 60% (Figure 4(h)). miR-342-3p mimic and anti-miR-342-3p markedly reduced or increased NFAM1 protein expression, respectively (Figure 4(i)). These findings indicate that miR-342-3p may inhibit NFAM1 expression by targeting its 3′-UTR. We then analyzed whether circITGB1 regulates NFAM1 expression. The knockdown of circITGB1 with siRNA remarkably inhibited NFAM1 expression, whereas anti-miR-342-3p antagonized the suppression of the NFAM1 expression level (Figure 4(j)). The overexpression of circITGB1 augmented the NFAM1 levels, and circITGB1 counteracted the inhibitory effect of miR-342-3p on NFAM1 expression (Figure 4(k)). These results indicate that circITGB1 functions as a miR-342-3p sponge, regulating NFAM1 expression in mdDCs.

### 3.5. circITGB1 Regulates mdDC Maturation through Targeting miR-342-3p and NFAM1

**Figure 5:**
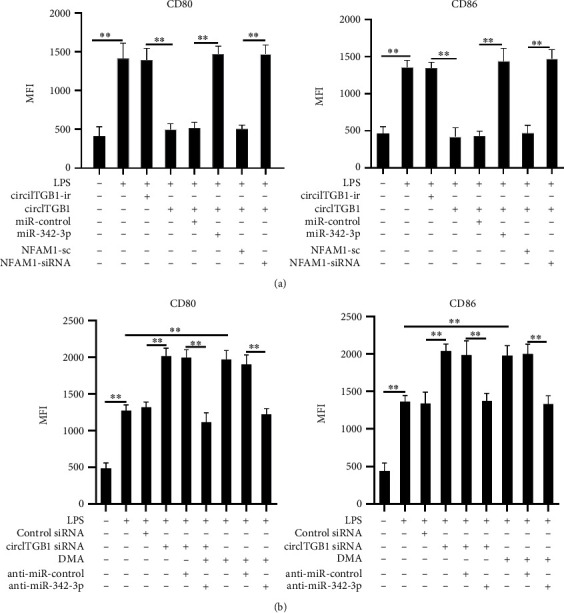
EV-circITGB1 regulates mdDC maturation via miR-342-3p and NFAM1. (a) Human mdDCs were pretreated with circITGB1-ir, circITGB1, miR-control, miR-342-3p, NFAM1-sc, or NFAM1-siRNA for 1 h prior to overnight stimulation with LPS (100 ng/mL). Cells were collected, stained, and analyzed for surface marker expression using flow cytometry. Bar graphs show expression of CD80 and CD86 on viable CD11c+ cells in 4 donors. Median fluorescence intensity of gated cells for corresponding surface markers ± SD, from 4 independent cultures. (b) Human mdDCs were pretreated with control siRNA, circITGB1 siRNA, anti-miR-control, anti-miR-342-3p, or DMA (21.3 mM) for 1 h prior to overnight stimulation with LPS (100 ng/mL). Cells were collected, stained, and analyzed for surface marker expression using flow cytometry. Bar graphs show expression of CD80 and CD86 on viable CD11c+ cells and are representative of 4 donors. Median fluorescence intensity of gated cells for corresponding surface markers ± SD, from 4 independent cultures. ^∗∗^*P* < 0.01.

We next explored whether miR-342-3p/NFAM1 are the downstream mediators of circITGB1 in mdDC maturation. The overexpression of miR-342-3p and knockdown of NFAM1 counteracted the inhibitory effect of circITGB1 on mdDC maturation as CD80 and CD86 expression increased (Figure 5(a)), whereas anti-miR-342-3p markedly reduced CD80 and CD86 expression in the presence of circITGB1 siRNA or EV release inhibitor DMA (21.3 mM) (Figure 5(b)). These results indicate that circITGB1 regulates mdDC maturation through the combined regulation of miR-342-3p and NFAM1.

### 3.6. EV-circITGB1 Regulates Cardiac Injury *In Vivo*

**Figure 6:**
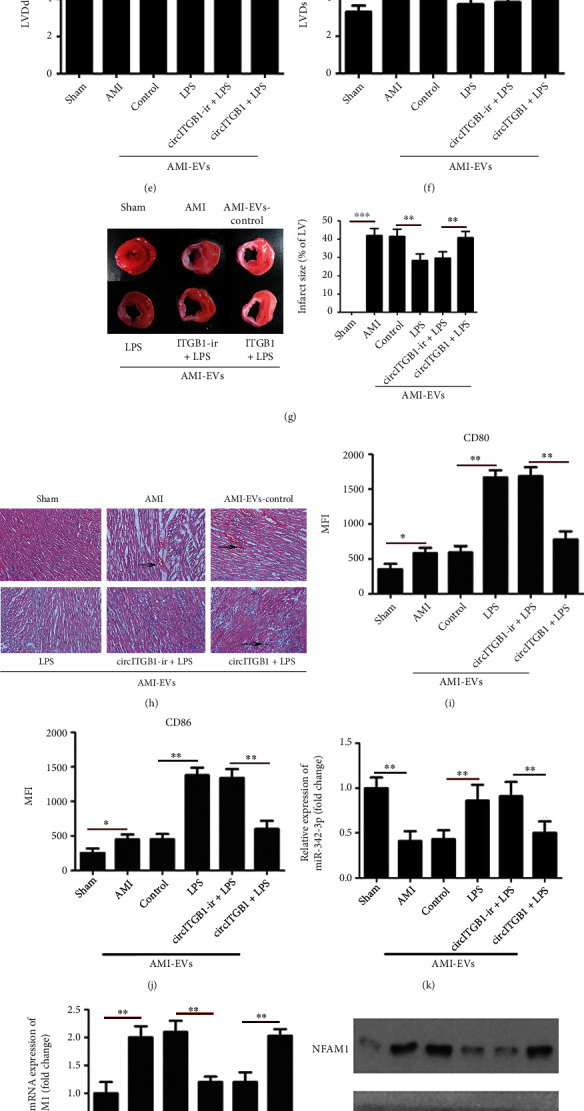
EV-circITGB1 regulates cardiac injury *in viv*o. (a) Relative expression of circITGB1 in EVs from AMI and healthy mice measured by qPCR; GAPDH was measured as a reference gene. Bone marrow-derived dendritic cells (BMDCs) were pretreated with circITGB1-ir and circITGB1 for 1 h prior to overnight stimulation with LPS, or without stimulation (control group). The EVs were isolated and injected into mice subjected to acute myocardial infarction (AMI) via the tail vein. EV-circITGB1 level (GAPDH was measured as a reference gene) (b), LV ejection fraction (EF) (c), the fractional shortening (FS) (d), and the left ventricular end-diastolic diameter (LVDd) (e) and end-systolic diameter (LVDs) (f) were measured by echocardiography at 4 weeks after AMI. (g) Representative images of 2,3,5-triphenyltetrazolium- (TTC-) stained myocardial tissues at 4 weeks after AMI in different-treated mice and quantification of the infarct size. Data are expressed as the means ± SD (*n* = 6). (h) Histological images with hematoxylin-eosin (H&E) staining of the myocardium (scale bar = 50 *μ*m) in the experimental groups studied; the arrows indicate infiltration of inflammatory cells. (i, j) Mean fluorescence intensity (MFI) of CD80 and CD86 after gating on CD11c+ in normal and different-treated mice was illustrated as bar graphs, expressing as means ± SD of 6 mice in each group. (k) qPCR detected miR-342-3p levels; U6 was measured as a reference gene. (l–n) qPCR and western blotting analyzed NFAM1 expression, GAPDH was measured as a reference gene, and *β*-actin was used as the loading control in western blotting. Significance was analyzed using Student's *t*-test or one-way ANOVA. ^∗^*P* < 0.05, ^∗∗^*P* < 0.01.

To assess the impact of EVs overexpressing circITGB1 on AMI *in vivo*, we used a surgical procedure to model AMI in mice. We measured the level of circITGB1 in plasma EVs of mice and found that circITGB1 was significantly increased in plasma EVs of AMI mice (Figure 6(a)). The fact that human and mouse cells have the same miR-342-3p sequence is interesting. The mouse NFAM1 is also a possible target of mmu-miR-342-3p (Figure S1A). As a next step, we then carried out a luciferase reporter assay using mmu-miR-342-3p mimics cotransfected with the luciferase reporter vectors (containing wt or mut mouse NFAM1 sequence) into HEK-293T cells. There was a big difference in the luciferase activity when the mmu-miR-342-3p mimics were used in cells with the wt NFAM1 sequence, but not in cells with the mut NFAM1 sequence (Figure S1B). BMDCs were pretreated with circITGB1-ir or circITGB1 for 1 h prior to overnight stimulation with or without LPS stimulation. EVs were injected into mice via the tail vein. As shown in Figure 6(b), we first determined the level of EV-circITGB1 expression in each group. Decreased FS and EF and increased LVDd and LVDs were observed after 4 weeks in the AMI mouse model compared with the sham-operated group. The injection of EVs from BMDCs stimulated with LPS increased FS and EF and decreased LVDd and LVDs, whereas circITGB1 counteracted the effect (Figures 6(c)–6(f)). The injection of EVs from BMDCs stimulated with LPS significantly decreased myocardial infarct size (MIS) compared to the AMI control 4 weeks after AMI. However, the MIS percentage was markedly increased in the circITGB1-treated groups compared to the LPS-treated groups (Figure 6(g)). In the present study, compared to the sham group, H&E staining indicated that the AMI and AMI-EV-control group demonstrated obvious histological damage in the infarct and border zone areas, presented as the appearance of necrotic tissue and increased irregularity, and infiltration of inflammatory cells. However, the areas of necrosis were limited, and the myocardium was orderly arranged in the LPS and circITGB1-ir+LPS groups; conversely, compared to the LPS group, the circITGB1+LPS group exhibited both focal, diffuse damage and infiltration of inflammatory cells in their myocardial structure (Figure 6(h)). Alongside, we sorted CD11c+BMDCs from different-treated mice and tested their activation in terms of costimulatory molecule expression of CD80 and CD86. Despite the elevation of the surface markers, circITGB1-treated groups were sufficient to suppress the expression of these costimulatory molecules on the BMDCs of AMI mice (Figures 6(i) and 6(j)). The injection of EVs from BMDCs stimulated with LPS increased miR-342-3p levels and decreased NFAM1 expression, whereas circITGB1 counteracted the effect (Figures 6(k)–6(n)). These results indicated that circITGB1 may aggravate cardiac injury via regulating miR-342-3p and NFAM1 expression *in vivo*.

## 4. Discussion

Several studies have implicated circRNAs in the progression of CVDs [19, 25, 38]. By transmitting a wide array of bioactive molecules, including proteins and miRNAs, EVs are regarded as crucial participants in long-range communication across the cardiovascular system. As a result, it has been demonstrated that these highly heterogeneous nanosized vesicles play a role in the maintenance of homeostasis of both the heart and vessels as well as in the pathophysiology of CVDs, making them potentially useful tools for the diagnosis, prognosis, and treatment of a variety of CVDs [39]. Therefore, in the present study, we compiled EV-circRNA expression profiles from the plasma of patients with AMI. We discovered a variety of differentially expressed circRNAs and chose circITGB1 for further investigation since it was consistently and significantly more expressed in patients with AMI as compared to matched healthy controls. By using luciferase screening and a biotin-labeled probe, we identified that circITGB1 competitively binds to miR-342-3p and inhibits its expression, which in turn increases the expression of NFAM1. NFAM1 has a regulatory effect on B cell development [40]. NFAM1 was recently discovered in a study examining immune response signatures. Additionally, it was discovered that NFAM1 is involved in the innate immune response and DC activation in healthy participants who received the yellow fever-17D vaccination [30]. miR-342-3p, which was revealed to interact with NFAM1 in our work, has been found to be differently expressed in blood-based miRNA profiles of cardiac amyloidosis, dilated cardiomyopathies, and heart failure in CVD research studies [41–43]. In a heart failure model in mice, miR-342-3p was among four miRNAs that were significantly downregulated in plasma and was used successfully as a biomarker to assess the efficacy of azilsartan medoxomil, an angiotensin II type 1 receptor blocker [44].

DCs are also known to participate in CVDs and are associated with atherosclerosis, hypertension, and heart failure [45]. In fact, a subset of DCs is located exclusively in the human heart, and NFAM1 belongs to a cluster of genes that is specifically associated with BDCA1-positive DCs [46, 47]. Kofler et al. [48] found different levels of DC activity in different subsets of CVDs. They found a downregulation of immature (CD1a+) DCs in ST-elevation myocardial infarction (STEMI), non-STEMI, and coronary artery disease (CAD) patients and an upregulation of mature (CD86+) DCs in CAD patients. Following AMI, it has been hypothesized that the downregulation of circulating immature DCs, which has been observed in several studies, may be associated with an increase in DC migration into the myocardium, which could be a significant factor in the pathophysiology of myocardial injury following AMI, as previously suggested [49].

Exosome inhibitors like GW4869 or DMA were mostly used in previous investigations [50, 51]. A previous study showed that GW4869 could significantly enhance chondrocyte differentiation and maturation [52]. In the present study, silencing of circITGB1 or suppression of EV release by DMA therapy dramatically increased the expression of CD80, CD83, CD86, and HLR-DR maturation markers in mdDCs. This suggests that circITGB1 may regulate the maturation of mdDCs, and it also suggests that DMA treatment supports the maturation of mdDCs.

miR-342-3p levels were found to be lower in response to higher levels of circITGB1, which is consistent with our hypothesis that circITGB1 may act as a miR-342-3p sponge to regulate NFAM1 expression. This is also consistent with other studies that have looked at the regulation of circRNAs in CVDs [24, 26]. Some scientists, on the other hand, contend that only a few number of circRNAs are present in cells at a sufficient level to have a noticeable impact on the biological activity of circRNA as efficient microRNA sponges [53, 54]. It is possible, though, that the dysregulation of circRNA through physiological factors could lead to an elevation of levels which then instigates a disease pathology, including an elevated level of EVs, as found in this study. In this study, miR- miR-342-3p was proven to be a miRNA-targeting NFAM1 in mdDCs. The results showed that miR-342-3p targets NFAM1's 3′-UTR and negatively regulates NFAM1 expression. The siRNA knockdown of circITGB1 significantly reduced NFAM1 expression, but anti-miR-342-3p reversed the effect. circITGB1 overexpression increased NFAM1 levels and counteracted the inhibitory effect of miR-342-3p on the NFAM1 expression. circITGB1 acts as a miR-342-3p sponge, regulating NFAM1 expression in mdDCs. miR-342-3p overexpression and NFAM1 knockdown counteracted circITGB1's maturation inhibitory impact. These findings show that circITGB1 regulates mdDC maturation via miR-342-3p and NFAM1. Recent evidence indicates that DCs play a significant role in the pathophysiological pathways behind a variety of cardiovascular illnesses, most notably AMI [45]. DCs are required for the recruitment and activation of immune cells, particularly T cells and macrophages, in the infarcted myocardium, which is followed by a significant increase in inflammatory cytokines [55]. After AMI in mice, DC infiltration is greatly increased in the infarcted area, and the percentage of mature DC-secreting exosomes is dramatically raised among cardiac DCs, which is attributable to the wounded cardiomyocytes' post-AMI milieu [56]. Our previous study indicated that exosomes generated from DCs improve heart function during AMI by activating CD4+ T lymphocytes [36]. Exosomes produced from stem cells have been shown to have cardiac therapeutic effects mostly through the gene products and microRNAs they contain, which induce angiogenesis, decrease cell death, and improve heart function [57]. circRNAs are a particular class of noncoding RNAs that have the ability to influence gene expression and modulate cell fate over a long period of time. Ischemic myocardial damage and myocardial remodeling have both been reported to be mediated by these extracellular circRNAs [58]. Our current study showed that circITGB1 significantly inhibited miR-342-3p expression and that circITGB1 exacerbated cardiac injury and dysfunction *in vivo*. Consistent with previous studies, for example, it has been shown that the circular RNA ciRs-126 may promote hypoxic/reoxygenated heart injury via miR-21 [59].

**Figure 7:**
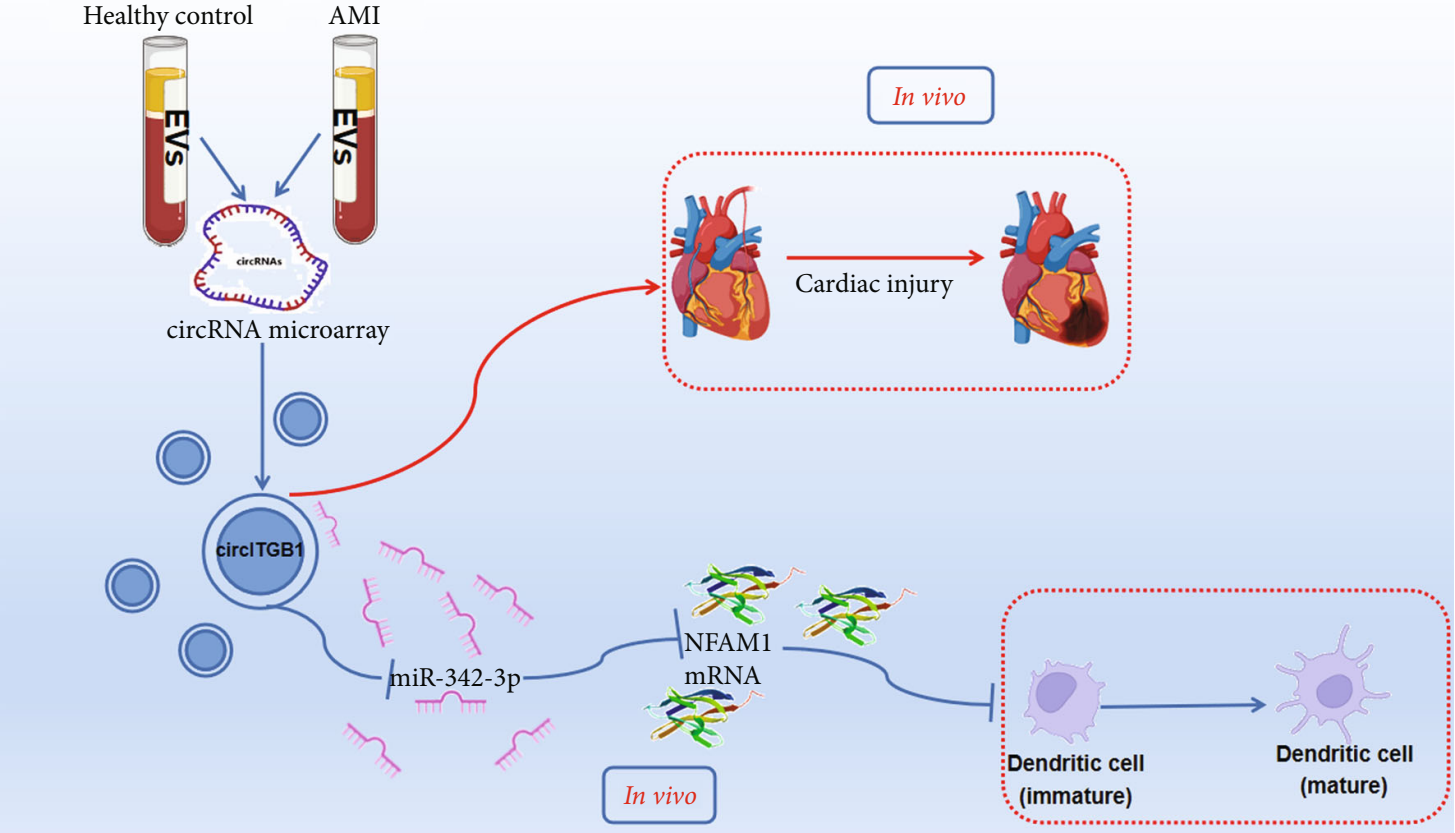
Model of EV-circITGB1 regulates maturation of dendritic cells (DCs). EV-circITGB1 acts as a miR-342-3p sponge to regulate NFAM1 expression, which adjusts the expression of DC maturation markers CD80 and CD86.

There are certain limitations to the current study. Despite the fact that we have established the first EV-circRNA expression profile in patients with AMI, our study was constrained by a small sample size and should be replicated on a bigger scale in the future. Furthermore, CVDs appears to be influenced by genetic background; hence, an expression profile from a larger demographic sample of patients would be advantageous. To summarize, we discovered that EV-circITGB1 affects DC maturation, presumably by functioning as a miR-342-3p sponge to modulate the expression of the nuclear factor adenovirus type 1 (NFAM1) (Figure 7). In addition, *in vivo* investigations have shown that circITGB1 exacerbates cardiac damage and dysfunction. EV-circITGB1 is implicated in DC maturation and cardiac damage, according to these findings, and it may have potential as a pharmacological target or biomarker for AMI.

## Figures and Tables

**Table 1:** Baseline clinical characteristics of patients and controls.

Variable	AMI (*n* = 20)	Control (*n* = 20)	*P* value (AMI vs. control)
Male	11 (55.0%)	10 (50.0%)	0.471
BMI (kg/m^2^)	23.5 ± 3.1	23.3 ± 2.6	0.583
Ages (years)	64.6 ± 11.3	63.1 ± 10.5	0.631
Smoke	12 (60.0%)	13 (65.0%)	0.468
Hypertension	9 (45.0%)	8 (40.0%)	0.421
Diabetes	5 (25.0%)	4 (20.0%)	0.163
Dyslipidemia	11 (55.0%)	10 (50.0%)	0.492
TC (mmol/L)	4.3 ± 0.91	3.90 ± 0.83	0.357
TG (mmol/L)	1.51 ± 0.93	1.42 ± 0.82	0.414
LDL-C (mmol/L)	2.50 ± 0.79	2.31 ± 0.68	0.277
HDL-C (mmol/L)	1.15 ± 0.98	1.24 ± 0.81	0.523
Lp (a) (mg/L)	225.12 ± 133.71	213.25 ± 121.43	0.428
cTnl (ng/mL)	21.8 ± 8.84	0	<0.001
Medications			
ACEI	15 (75.0%)	—	—
ARB	16 (80.0%)	—	—
Statin	18 (90.0%)	—	—
Dual antiplatelet	18 (90.0%)	—	—

AMI: acute myocardial infarction; BMI: body mass index; TC: total cholesterol; TG: triglyceride; LDL-C: low-density lipoprotein cholesterol; HDL-C: high-density lipoprotein cholesterol; Lp (a): lipoprotein (a); ACEI: angiotensin converting enzyme inhibitors; ARB: angiotensin receptor blockers. Categorical data are provided as frequency with percentages, and comparisons between categorical variables were conducted utilizing the chi-square test. Normally distributed quantitative data are expressed as mean ± SD, nonnormal as median and interquartile range (IQR). Comparisons between quantitative variables were done using unpaired Student's*t*-test. Consecutive measurements were evaluated with the paired *t*-test.

## Data Availability

The related data during the present study are available from the corresponding author on reasonable request.

## Supplementary Materials

Supplementary Materials Supplementary Figure S1: NFAM1 is a target of mmu-miR-342-3p in mouse. (A) A putative binding site of mmu-miR-342-3p with respect to NFAM1 was predicated via TargetScan. (B) The luciferase activity of pLG3-NFAM1 in HEK-293T cells after cotransfection with mmu-miR-342-3p. Unpaired Student's *t*-test was used for the statistical analyses. ∗∗*P* < 0.01. Supplementary Table S1: primers used for qPCR assay.
