# Supplementary material for: Extracellular Vesicle-Derived circITGB1 Regulates Dendritic Cell Maturation and Cardiac Inflammation via miR-342-3p/NFAM1
Source: Oxid Med Cell Longev. 2022 May 16;2022:8392313. doi: 10.1155/2022/8392313 (PMC9126660; doi:10.1155/2022/8392313)
Supplement: Supplementary Materials — Supplementary Figure S1: NFAM1 is a target of mmu-miR-342-3p in mouse. (A) A putative binding site of mmu-miR-342-3p with respect to NFAM1 was predicated via TargetScan. (B) The luciferase activity of pLG3-NFAM1 in HEK-293T cells after cotransfection with mmu-miR-342-3p. Unpaired Student's t-test was used for the statistical analyses. ∗∗P < 0.01. Supplementary Table S1: primers used for qPCR assay. [file 8392313.f1.zip › Figure S1.pdf]

A

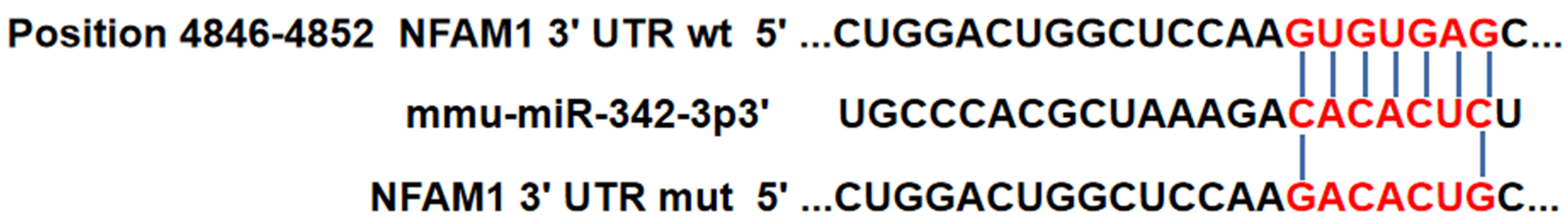
Position 4846-4852 NFAM1 3' UTR wt 5' ...CUGGACUGGCUCCAAGUGUGAGC...
mmu-miR-342-3p3' UGCCCACGCUAAAGACACACUCU
NFAM1 3' UTR mut 5' ...CUGGACUGGCUCCAAGACACUGC...

B

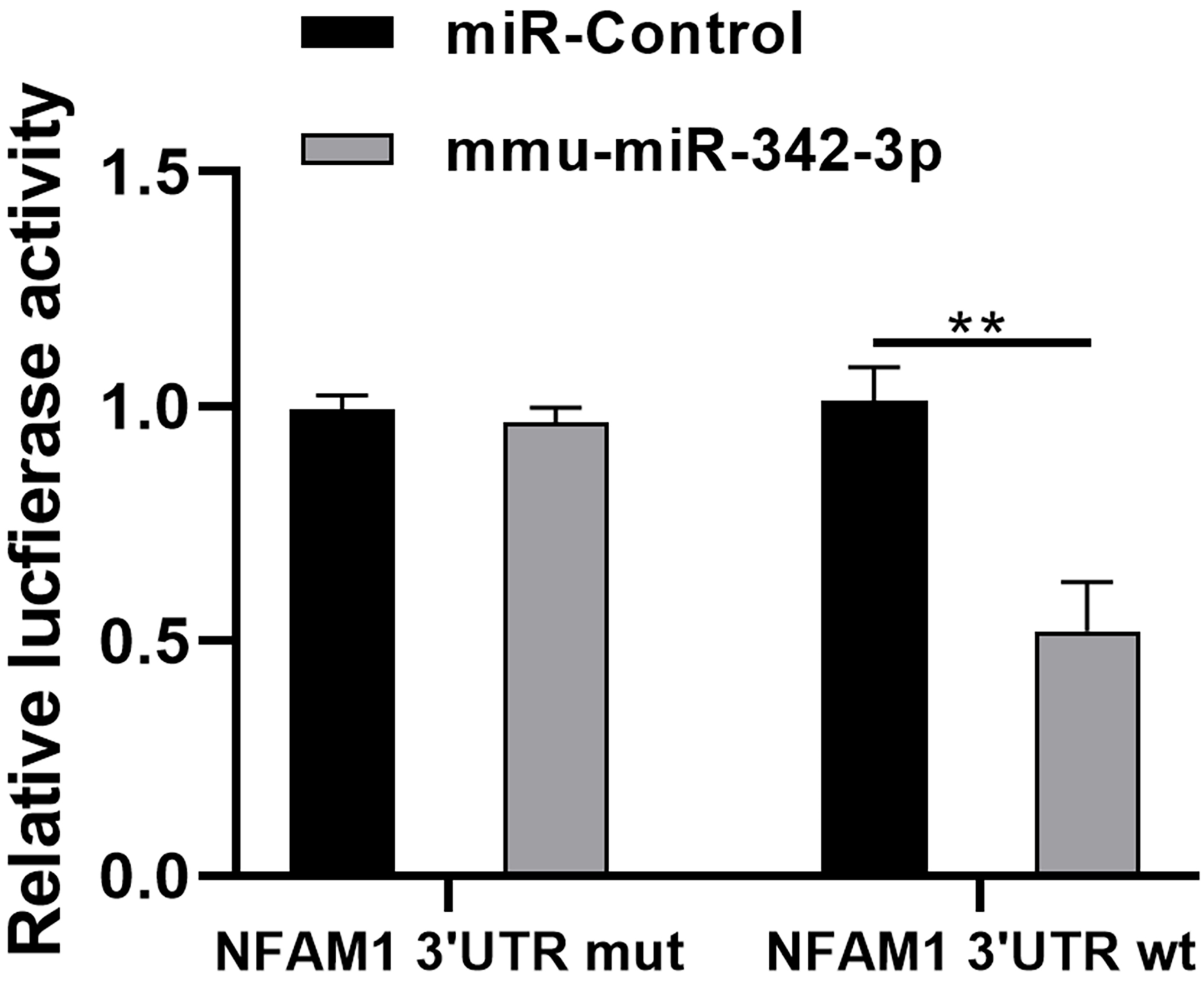
miR-Control
mmu-miR-342-3p
Relative lucfierase activity
1.5
1.0
0.5
0.0
**
NFAM1 3'UTR mut
NFAM1 3'UTR wt
