# Supplementary material for: Extracellular Vesicle-Derived circITGB1 Regulates Dendritic Cell Maturation and Cardiac Inflammation via miR-342-3p/NFAM1
Source: Oxid Med Cell Longev. 2022 May 16;2022:8392313. doi: 10.1155/2022/8392313 (PMC9126660; doi:10.1155/2022/8392313)
Supplement: Supplementary Materials — Supplementary Figure S1: NFAM1 is a target of mmu-miR-342-3p in mouse. (A) A putative binding site of mmu-miR-342-3p with respect to NFAM1 was predicated via TargetScan. (B) The luciferase activity of pLG3-NFAM1 in HEK-293T cells after cotransfection with mmu-miR-342-3p. Unpaired Student's t-test was used for the statistical analyses. ∗∗P < 0.01. Supplementary Table S1: primers used for qPCR assay. [file 8392313.f1.zip › Supplementary materials (2).docx]

**Supplementary materials**


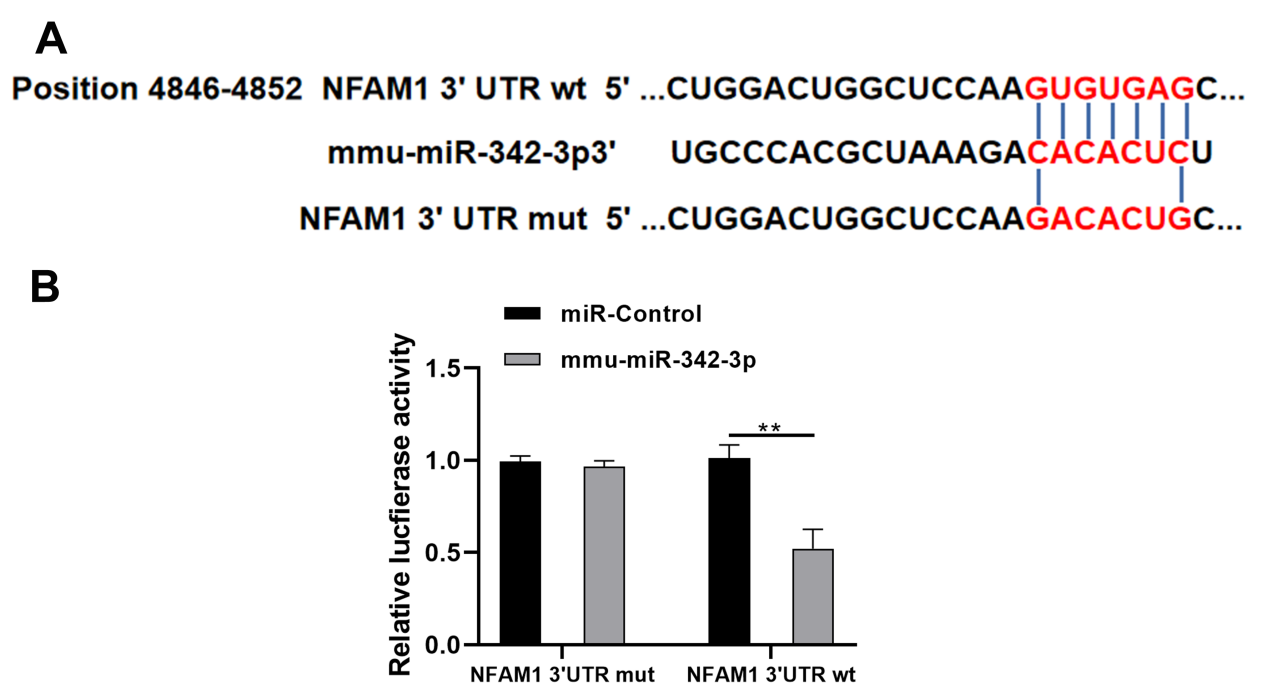


Figure legend

**Figues S1.** **NFAM1 is a target of mmu-miR-342-3p in mouse.** (**A**) a Putative binding site of mmu-miR-342-3p with respect to NFAM1 was predicated via Targetscan. (**B**) The luciferase activity of pLG3-NFAM1 in HEK-293T cells after co-transfection with mmu-miR-342-3p. Unpaired student’s t-test was used for the statistical analyses. **p<0.01.

**Table S1. Primers used for qPCR assay.**

| Gene | Forward primer (5’-3’) | Reverse primer (5’-3’) |
| --- | --- | --- |
| circITGB1 | TCGGGACAAATTACCCCAGC | GTTGCACTCACACACACGAC |
| circSlc7a1 | GACGAGCTGATAGGCAGACC | TAGAGGTAAGCTGAGCCCGT |
| circCOL4A1 | CGTGCACAGCCAGACCATTC | CTCCTCTTTCACCAGGGAAG |
| circSIDT2 | AGAAAACCCCTGCAGAGTCG | AGCACCTGGAGCTTACCAAG |
| circTIMM44 | AGCCAAGCCACTTTAGGTCC | GAACAGGCCCCTGCTCTTCT |
| circCRIP1 | AACACTTGGAATCTCGCGCC | CCTCCTTGTTGCACTTGGGA |
| circNUP188 | GACATTGCATGCTCTGTGGCA | GCACCATCACTCTGATGTCTC |
| circACVR1 | GATGACCTGTAAGACATGC | TGGGCTTCTCATCTTTAGTG |
| circUSP53 | CACTAGCCCCTACCAAAGGC | TCTTTAACAGGACGAAGGGT |
| circNNT | CAACTTCTCAGGGCCTTGCT | TCGAACAATTGCACCCATCG |
| circAP2M1 | AAGCAAGAGGGCGGCGCATA | CGCTCTGAGAACAGACCTGG |
| circNBEAL1 | TGGAACAAATCAGCATTGAC | AACATGACGAATCTCAAGA |
| circTTC13 | CAGAGGACTTCTGAAGGAAGC | CTTGGGTTCGCAGTCTGAGT |
| circTCERG1 | TTACCTTTCTGGGTGTTTCTTGT | TCCATCGAGACTGACTGTCCA |
| circSHC3 | AGAAGGCACGAATGCAGAGT | ATGGGGTCTGGGTTTCAGTC |
| circZNF700 | GCCTTCAGATCTGCCAAGATT | ACGACCATCTCCATTTCCGC |
| circGRN | CCGAGACCGTCCACTACAAG | ACGTAGGGAAACTCGTCATCC |
| circUBA2 | TGGGCCATCAATCGGAAACT | AGATGGACAGTGCAGAAGGTC |
| circATG5 | GCAACTCTGGATGGGATTGC | AGATGGACAGTGCAGAAGGTC |
| circPOLR1A | TACCAGCTGCGGTTTCAGTT | GGAGCTTCACTGTCACCTCTT |
| miR-342-3p | GGGTCTCACACAGAAATCGC | CAGTGCGTGTCGTGGAGT |
